# Estimation and projection of the national profile of cancer mortality in China: 1991–2005

**DOI:** 10.1038/sj.bjc.6601813

**Published:** 2004-04-27

**Authors:** L Yang, D M Parkin, L D Li, Y D Chen, F Bray

**Affiliations:** 1 International Agency for Research on Cancer, 150, cours Albert Thomas, 69372 Lyon, Cedex 08, France; 2 National Office for Cancer Prevention and Control, 17 South Lane, Panjiayuan, Chaoyang District, Beijing 10021, PR China; 3 Center of Health Information and Statistics of Ministry of Health/Public Health School, Peking University, 38 Xueyuan Road, Beijing 100083, PR China

**Keywords:** mortality, model, projections, China

## Abstract

There are no national-level data on cancer mortality in China since two surveys in 1973–1975 and 1990–1992 (a 10% sample), but ongoing surveillance systems, based on nonrandom selected populations, give an indication as to the trends for major cancers. Based on a log-linear regression model with Poisson errors, the annual rates of change for 10 cancers and all other cancers combined, by age, sex and urban/rural residence were estimated from the data of the surveillance system of the Center for Health Information and Statistics, covering about 10% of the national population. These rates of change were applied to the survey data of 1990–1992 to estimate national mortality in the year 2000, and to make projections for 2005. Mortality rates for all cancers combined, adjusted for age, are predicted to change little between 1991 and 2005 (−0.8% in men and +2.5% in women), but population growth and ageing will result in an increasing number of deaths, from 1.2 to 1.8 million. The largest predicted increases are for the numbers of female breast (+155.4%) and lung cancers (+112.1% in men, +153.5% in women). For these two sites, mortality rates will almost double. Cancer will make an increasing contribution to the burden of diseases in China in the 21st century. The marked increases in risk of cancers of the lung, female breast and large bowel indicate priorities for prevention and control. The increasing trends in young age groups for cancers of the cervix, lung and female breast suggest that their predicted increases may be underestimated, and that more attention should be paid to strategies for their prevention and control.

Comparison of two representative surveys of mortality in China (in 1973–1975 and 1990–1992) (NOCPC, 1979; Li *et al*, 1996) shows that overall cancer mortality has increased, and that the trends differ depending on the type of cancer and urban/rural area of residence (Li *et al*, 1996). An accurate knowledge of cancer patterns and their likely evolution in the future are essential in the planning of national cancer control programmes (WHO, 2002). However, the absence of national statistics on mortality or incidence of cancer in China (Yang *et al*, 2003a) means that estimation procedures, using available ongoing surveillance systems, are required.

The most comprehensive data on mortality in China are those from the continuing surveillance system, involving 36 cities and 85 counties, that was conducted by the Center for Health Information and Statistics (CHIS). This system covers a population of about 100–120 millions (about 10% of the Chinese population), and is used by WHO to report mortality rates for the urban and rural populations of China (Cancer Mortality Database, 2003; Yang *et al*, 2003a).The surveillance areas do not represent a random sample of the Chinese population, but are located in the more easily accessible cities and counties, on the eastern seaboard, where vital statistics systems are relatively well developed (Yang *et al*, 2003a). As a result, the mortality rates are not the same as those in the representative national survey, although after appropriate weighting by age, sex and urban–rural status show a close approximation for many sites (Yang *et al*, 2004: in press). The trends in mortality within such strata are probably representative of those at the national level. We use observed trends since 1991, and the data from the national mortality survey of 1990–1992, to estimate cancer mortality rates in China in 2000, and to prepare projections to the year 2005. These rates, combined with knowledge of expected changes in the size and age structure of the population, allow estimation of current and future numbers of cancer deaths at the national level.

## MATERIAL AND METHODS

### Material

The following sources of data were used:

1. Population details, by sex, age group and urban/rural residence from the 1990 and 2000 censuses (Population Census Office, 1993, 2002).
2. The projected population in 2005, by age and sex, from the United Nations Population Division (UN Population Division, 2003);
3. The estimated urban : rural ratios of the total population in 1995, 1998, 1999 and 2000 (from census data) (Population Census Office, 2002; UN Statistics Division, 2002a); and
4. Cancer mortality rates, by cause (10 sites), sex, age group and urban/rural residence from the second National Mortality Survey in 1990–1992 (Li *et al*, 1996; Zhou *et al*, 1997), and for each year (1991–1999) from the population covered by the cancer reporting system of CHIS (Cancer Mortality Database, 2003);

### Population estimation and projection

The annual change in the proportion of the total population classified as rural was calculated from the UN population estimates from 1995 to 1999 (UN Statistics Division, 2002a) and 2000 census data (Population Census Office, 2002). The exponential of this change was applied to estimate the proportion of the total population in 2005 having rural residence (and, by subtraction, urban). This proportion (all ages) was used to obtain the proportion in individual age groups, by multiplying the ratio of % rural in the age group concerned with the % rural of all ages in 2000. These proportions were used to calculate the population numbers by age, sex and urban and rural residence, from the UN population projections (totals by age and sex) for 2005.

### Estimates and projections of cancer mortality rates and numbers of deaths

Mortality rates for each year (1991–1999) for 10 specific cancer sites, oesophagus, stomach, lung, liver, colon-rectum, female breast, cervix uteri, nasopharynx, leukaemia and bladder, are available in the CHIS data set (Cancer Mortality Database, 2003). The estimated annual percent changes (EAPCs) in the rate for these cancers, as well as all other cancers combined, for this 9-year period were calculated by area (rural/urban), sex and age group (0–4, 5–14, 15–44, 45–54, 55–64, 65–74, 75+ years), based on a Poisson regression model. These rates of change were applied to the mortality rates observed in the national mortality survey in 1990–1992 to estimate the age-specific mortality rate at the national level for the corresponding cancers from 1991 to 2005. The model used was:


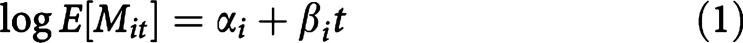


where *E*[*M*_*it*_] is the expected mortality rate of the cancer in age group *i* and year *t*, *α*_*i*_ is the baseline age-specific mortality rate (when *t*=0 e.g. 1991) and *β*_*i*_ is the observed EAPC in the CHIS data for the age group *i* (Hakulinen and Dyba, 1994).

With the exception of leukaemia, we assumed that mortality remained constant in the youngest age group (less than 15 years old), because of the uncertainty of the calculated EAPCs, which were based on small numbers of deaths in this age group. The expected numbers of deaths for each cancer, by age group, sex and urban/rural residence were then calculated using the population estimates for 1991–2005, estimated as described above. The numbers were then summed for urban–rural categories, and age-specific rates calculated for the whole population. Age-standardised rates (ASRs) were calculated for years 1991, 2000 and 2005 using the weights of the world standard population. The numbers of deaths and mortality rates for all cancers combined were calculated by summing the estimates for the 11 categories (10 specific cancer sites, plus ‘other cancers’).

The changes in the numbers of deaths between 1991 and 2000 or 2005 are due to changes both in the risk of death from cancer and in the size and structure of the population. We calculated the component due to risk as the difference between the estimated future deaths, and the number that would have occurred if the mortality rates had remained the same as in 1991. The change due to population size and ageing is obtained as the difference between this latter figure, and the original number of deaths (in 1991) (Engeland *et al*, 1993).

### Sensitivity analysis

Other models have been suggested for making predictions of future rates of disease, assuming Poisson-distributed counts in strata defined by age and sex. In addition to the model employed in this paper, Hakulinen and Dyba (1994) propose two other models:


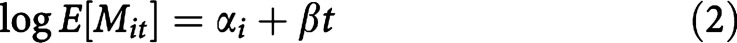



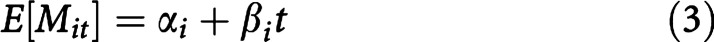


Model (2) assumes the same proportional log-linear changes over time within age groups, while model (3) allows different linear changes on an arithmetic scale among different age groups. The log-linear model is usually chosen for cancers with decreasing or stable trends, as it avoids prediction of negative rates, whereas model (3) is useful for modelling increasing trends, as it avoids an overestimation of the prediction often produced in models (1) and (2). Dyba *et al* (1997) also proposed a simple nonlinear model:


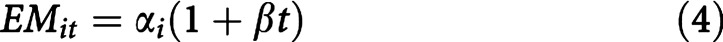


This model assumes proportional effects for different age groups, but with the constraint that the absolute change in age-specific mortality is proportional to the corresponding baseline rates (*α*_*i*_), and so that the shape of the age-incidence curve remains unchanged.

To validate our choice of prediction model, each of the four models was fitted to compare observed and expected numbers of deaths, age-specific and age-standardised rates of cancers of the lung (in rural females), breast (in rural females), oesophagus (in urban females) and cervix cancer (in urban females) in 1999. Since the small numbers of observations may produce unrealistic increasing or decreasing trends for particular age groups, the younger age groups (age 0–44) were combined.

## RESULTS

### Estimated and projected population data

**Figure 1:**
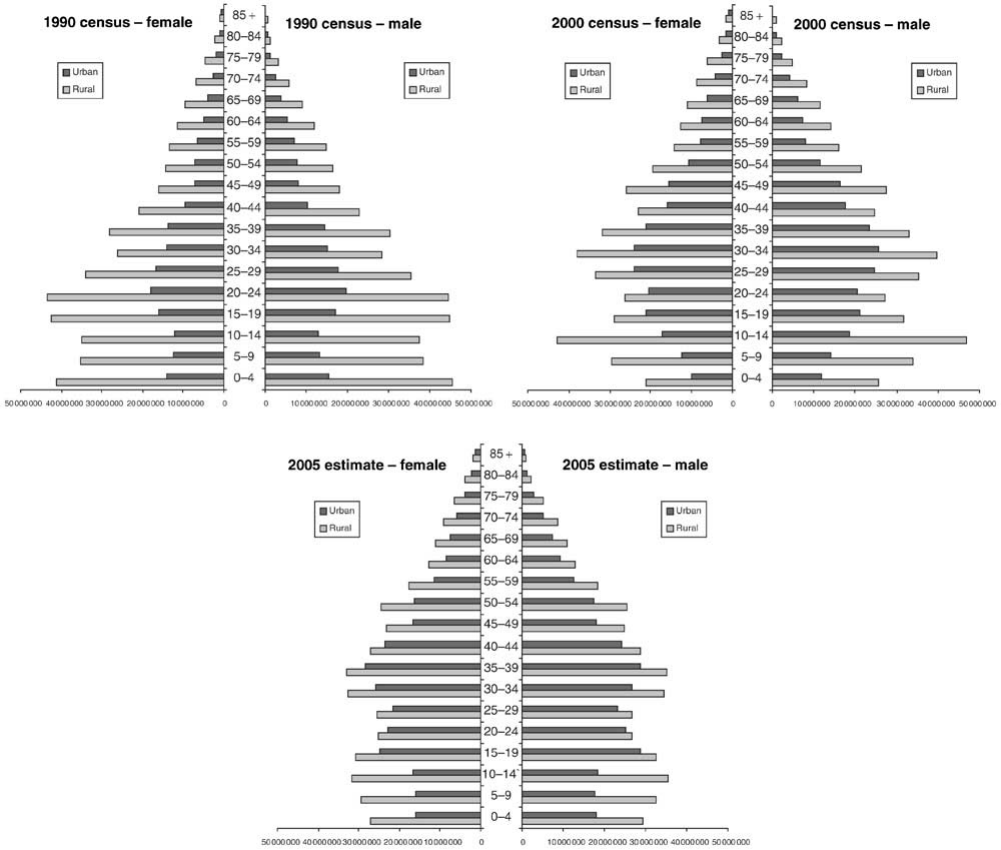
Population pyramids for the years 1990, 2000, 2005 by area, sex and age group in China.

Based on the latest population census, the total population of China was 1.24 billion (0.64 male and 0.60 female subjects) in 2000 and is projected to be 1.32 billion (0.68 male and 0.64 female subjects) in 2005 (Population Census Office, 2002; UN Population Division, 2003). Figure 1

shows the population pyramids for 1990, 2000 (from census data) and 2005 by sex, age and area. The proportion of the population in urban areas is increasing in each age group, while that of people aged over 60 years also increased, from 8.6% in 1990 to 10.5% in 2000, and 10.8% in 2005.

### Recent trends in cancer mortality rates, and estimated and projected numbers of deaths

The EAPCs of the mortality rates in the CHIS data set, by cancer site, age group, area and sex, are shown in Table 1

**Table 1:** Estimated annual percent changes (EAPCs) in mortality rates of cancer by site, age, area and sex

**Cancer**	**EAPC (age group) (years)**	**rural-male**	**rural-female**	**urban-male**	**urban-female**
Stomach	15–44	0.1	−0.9	0.7	0.0
	45–54	−0.6	−1.1	0.4	−0.5
	55–64	−2.7	−2.6	−4.2	−2.8
	65–74	−2.5	−1.9	−2.8	−2.9
	75+	−2.0	−2.6	−1.6	−2.2
					
Oesophagus	15–44	−2.8	−5.5	5.1	−0.1
	45–54	−2.0	−4.1	2.3	−4.3
	55–64	−3.8	−3.9	−4.0	−4.3
	65–74	−1.5	−3.3	−3.4	−3.9
	75+	−1.7	−1.7	−1.9	−2.4
					
Liver	15–44	0.5	2.6	2.0	2.1
	45–54	1.7	2.4	1.2	0.2
	55–64	−0.3	0.7	−2.2	0.4
	65–74	1.8	1.0	−0.3	0.6
	75+	2.0	0.8	0.3	1.1
					
Lung	15–44	4.4	2.4	4.1	3.0
	45–54	4.6	6.7	1.8	0.0
	55–64	0.5	4.1	−2.3	−0.5
	65–74	3.9	4.7	0.9	1.3
	75+	4.8	5.3	2.6	3.6
					
Colon-rectal	15–44	1.5	−1.5	−0.7	−0.9
	45–54	0.7	−0.5	3.6	2.0
	55–64	−0.3	−0.5	−0.5	−0.9
	65–74	2.1	−0.5	2.2	1.6
	75+	1.1	−2.2	1.5	1.3
					
Cervix	15–44	—	2.8	—	7.3
	45–54	—	2.7	—	4.2
	55–64	—	−3.1	—	−7.7
	65–74	—	−5.8	—	−9.4
	75+	—	−6.5	—	−7.6
					
Breast (female)	15–44	—	5.0	—	3.3
	45–54	—	7.3	—	2.8
	55–64	—	5.1	—	1.9
	65–74	—	4.0	—	0.1
	75+	—	0.0	—	−1.0
					
Leukaemia	0–4	−7.9	−8.3	0.5	−4.1
	5–14	2.6	2.0	−0.9	0.1
	15–44	−1.7	−1.9	−0.1	−1.4
	45–54	0.4	−0.7	2.7	1.9
	55–64	0.9	−2.3	−1.2	−0.9
	65–74	3.7	−2.1	1.3	0.0
	75+	6.1	1.7	2.9	0.8
					
Nasopharynx	15–44	−0.3	−1.0	−2.9	−3.4
	45–54	−1.0	3.4	−0.6	−3.9
	55–64	−1.1	−2.6	−4.8	−2.4
	65–74	1.4	1.7	−2.2	−2.2
	75+	−1.0	−2.1	0.5	0.0
					
Bladder	15–44	−2.4	8.7	4.1	−7.6
	45–54	1.8	3.5	3.5	4.0
	55–64	−1.0	3.3	−1.9	−1.4
	65–74	0.3	−3.4	−0.9	−3.3
	75+	−1.2	−2.9	0.1	0.0
					
Other Sites	15–44	−0.5	−0.6	0.7	1.7
	45–54	1.3	1.7	2.1	0.9
	55–64	0.1	−0.6	−1.9	−0.7
	65–74	1.8	0.3	0.2	1.9
	75+	1.1	−0.3	1.9	2.3

. The estimated and projected national age-specific and age-standardised mortality rates in 1991, 2000 and 2005 (prepared by combining the data for rural and urban areas) are shown in Table 2

**Table 2:** Age-specific and age-standardised cancer mortality rates (per 100 000) for year 1991, 2000 and 2005, by site and sex

		**Year 1991**						**Year 2000**						**Year 2005**					
		**Age groups (years)**						**Age groups (years)**						**Age groups (years)**					
**Cancer**	**sex**	**15–44**	**45–54**	**55–64**	**65–74**	**75+**	**WASR91**	**15–44**	**45–54**	**55–64**	**65–74**	**75+**	**WASR00**	**15–44**	**45–54**	**55–64**	**65–74**	**75+**	**WASR05**
All sites	Male	31.2	223.2	562.3	1042.7	1282.4	162.4	32.9	243.0	477.0	1048.9	1353.9	160.2	34.5	256.6	430.4	1066.4	1433.8	161.1
All sites	Female	20.0	126.2	283.3	531.0	678.4	86.6	21.3	141.4	263.0	515.8	673.9	86.3	22.7	154.0	257.8	522.4	701.0	88.8
Stomach	Male	4.0	48.7	145.8	293.0	344.6	40.3	3.9	45.5	109.4	229.4	289.6	32.7	3.9	43.3	89.4	198.3	261.9	28.8
Stomach	Female	2.7	23.1	60.7	129.2	167.3	18.4	2.6	20.5	46.6	104.6	133.3	15.0	2.6	19.0	40.1	91.3	116.3	13.3
Oesophagus	Male	2.1	31.4	96.5	203.5	248.4	27.2	1.7	27.0	66.9	167.8	210.0	21.6	1.7	25.0	52.0	147.8	188.5	18.8
Oesophagus	Female	1.1	16.6	47.7	97.0	118.6	13.4	0.7	11.0	32.1	69.1	99.1	9.5	0.5	8.5	25.6	55.7	86.6	7.7
Liver	Male	11.5	70.3	117.0	158.9	164.1	33.3	12.3	79.5	108.3	175.2	187.8	35.3	13.0	84.3	101.2	183.0	200.9	36.2
Liver	Female	3.2	21.2	41.8	68.6	79.0	12.1	3.8	24.5	43.6	73.8	85.8	13.3	4.2	26.2	44.5	76.4	89.4	13.9
Lung	Male	3.2	33.8	113.9	214.1	243.1	29.8	4.7	46.8	108.5	277.7	347.0	36.7	5.8	55.7	106.5	321.8	430.2	41.8
Lung	Female	1.8	16.6	42.1	78.4	93.1	11.8	2.3	24.8	52.1	107.7	142.4	16.1	2.6	30.9	58.6	128.9	183.4	19.3
Colorectal	Male	1.5	7.6	20.6	46.2	79.8	7.1	1.6	9.0	19.9	56.3	90.3	7.9	1.7	10.2	19.7	63.1	98.6	8.6
Colorectal	Female	1.3	6.6	14.5	32.8	55.3	5.2	1.2	7.1	13.9	34.8	52.8	5.2	1.2	7.7	13.6	37.3	55.3	5.4
Cervix	Female	0.9	7.8	13.9	26.1	28.5	4.2	1.2	10.0	9.4	14.0	15.4	3.4	1.4	11.3	7.5	9.7	10.9	3.2
Breast (female)	Female	1.6	9.2	12.5	14.4	20.8	3.8	2.4	15.2	17.9	18.3	20.2	5.5	3.0	20.1	21.8	20.8	20.5	6.7
Leukaemia	Male	3.4	4.5	6.5	8.8	8.9	4.1	3.0	5.0	6.7	11.5	13.8	4.2	2.9	5.5	6.7	13.3	17.8	4.4
Leukaemia	Female	2.9	4.0	5.4	6.3	5.4	3.4	2.5	4.1	4.6	5.8	6.1	3.0	2.3	4.2	4.3	5.7	6.7	2.8
Nasopharynx	Male	1.0	6.2	9.6	11.1	11.8	2.7	0.9	5.7	7.8	11.4	11.3	2.4	0.8	5.5	6.8	11.4	11.2	2.3
Nasopharynx	Female	0.5	2.4	4.0	5.1	6.5	1.2	0.4	2.7	3.2	5.4	5.7	1.1	0.4	2.8	2.8	5.4	5.4	1.1
Bladder	Male	0.1	1.1	4.3	13.9	35.7	1.9	0.1	1.3	3.9	14.0	34.4	1.9	0.1	1.5	3.6	14.1	35.1	1.9
Bladder	Female	0.1	0.5	1.3	4.3	8.4	0.6	0.1	0.7	1.5	3.3	7.5	0.6	0.1	0.8	1.7	2.9	7.6	0.6
Other sites	Male	4.5	19.7	48.1	93.2	146.0	16.1	4.6	23.1	45.5	105.6	169.7	17.3	4.6	25.5	44.6	113.6	189.7	18.4
Other sites	Female	3.9	18.2	39.4	68.8	95.5	12.6	4.1	20.8	38.1	78.9	105.5	13.6	4.4	22.5	37.4	88.2	118.9	14.6

, by site and sex. The numbers of deaths in 1991, 2000 and 2005, the percentage changes over time and the contributions due to changes in rates and changes in population (size and age structure) by site and sex are shown in Table 3

**Table 3:** Changes in the numbers of deaths between 2000 (2005) and 1991 and relative changes due to changed risk, population age structure and population size, by cancer site and sex

		**Year 1991**		**Year 2000**					**Year 2005**			
				**Change compared with 1991**		**Change (%) due to**			**Change compared with 1991**		**Change (%) due to**	
**Cancer**	**Sex**	**No. of deaths**	**No. of deaths**	**No.**	**%**	**Risk**	**Pop**	**No. of deaths**	**No.**	**%**	**Risk**	**Pop**
All sites	Male	751 921	992 585	240 664	32.0	−1.1	33.1	1 111 143	359 223	47.8	−0.3	48.1
All sites	Female	421 282	549 962	128 680	30.5	0.2	30.3	648 930	227 648	54.0	4.6	49.4
Stomach	Male	181 481	200 518	19 037	10.5	−24.7	35.2	197 222	15 741	8.7	−42.1	50.8
Stomach	Female	88 790	95942	7152	8.1	−23.9	31.9	97 843	9054	10.2	−41.9	52.1
Oesophagus	Male	121 508	131 794	10 285	8.5	−27.4	35.9	127 726	6218	5.1	−46.5	51.7
Oesophagus	Female	63 756	60 598	−3159	−5.0	−37.3	32.4	57 485	−6272	−9.8	−62.9	53.1
Liver	Male	162 004	225 486	63 483	39.2	8.3	30.9	256 679	94 675	58.4	13.1	45.3
Liver	Female	58 620	84 349	25 729	43.9	13.2	30.7	100 945	42 325	72.2	22.8	49.4
Lung	Male	134 748	224 807	90 058	66.8	32.5	34.4	285 785	151 037	112.1	62.1	50.0
Lung	Female	56 468	102 836	46 368	82.1	50.5	31.6	143 151	86 683	153.5	102.0	51.6
Colorectal	Male	32 290	48 869	16 579	51.3	16.6	34.7	58 553	26 263	81.3	31.6	49.8
Colorectal	Female	25 669	33 913	8244	32.1	0.5	31.6	40 429	14 761	57.5	6.0	51.5
Cervix	Female	20 197	21 461	1264	6.3	−25.9	32.2	22 975	2778	13.8	−37.7	51.4
Breast (female)	Female	18 857	34 927	16 070	85.2	54.8	30.4	48 164	29 307	155.4	107.5	47.9
Leukaemia	Male	22 861	26 585	3724	16.3	3.2	13.1	29 640	6779	29.7	8.1	21.6
Leukaemia	Female	17 996	18 322	327	1.8	−10.7	12.5	19 094	1098	6.1	−16.6	22.7
Nasopharynx	Male	13 207	15 670	2463	18.7	−11.5	30.2	16 400	3193	24.2	−20.3	44.5
Nasopharynx	Female	5819	7095	1276	21.9	−7.4	29.3	7802	1983	34.1	−12.8	46.9
Bladder	Male	8203	11 417	3214	39.2	−1.5	40.7	12 935	4732	57.7	0.6	57.1
Bladder	Female	2845	3675	830	29.2	−4.5	33.7	4430	1585	55.7	0.4	55.3
Other sites	Male	75 619	107 439	31 821	42.1	10.9	31.2	126 204	50 585	66.9	21.5	45.4
Other sites	Female	62 264	86 844	24 579	39.5	11.0	28.5	106 611	44 347	71.2	24.5	46.7

.

The figures in appendix A show the observed trends in age-standardised mortality rates for 10 cancers, and for the other cancers combined, by site, area and sex, based on the CHIS surveillance results in 1991–1999 (symbolised as ‘Rural-O’ or ‘Urban-O’). The age-standardised mortality rates in 1991, based on the national mortality survey in 1990–1992, are also shown (the first point of the lines labelled as ‘Rural-E’ or ‘Urban-E’), together with the expected evolution of this rate from 1992 to 2005 (the remaining points on the lines), based on the EAPCs calculated from the CHIS data. The estimated age-standardised mortality rates for cancers of the oesophagus, stomach (in rural populations), lung (in rural female subjects) and cervix (in urban female subjects) are somewhat higher than those observed in the CHIS populations, while they are lower for cancers of the lung (in urban females), colon-rectum (except in rural males), breast (in urban females), bladder (in urban males) and other sites combined (in urban populations).

During the study period, there were large increases in age-standardised mortality rates of cancers of lung and breast, and to a lesser extent, of liver, among all populations and among almost all age groups (Table 2 and Appendix A). The dramatic increases in the rates in populations younger than age 55 years if correct imply that there will be further increases in risk from these cancers in future. The increasing trends are much steeper among rural populations, so that mortality rates from cancers of the lung and breast are projected to reach the same levels as those in urban areas by 2005, although they were considerably lower in rural populations in 1991 (Appendix A). Along with population growth and ageing, the escalation in mortality rates means that the component of change in deaths due to risk is large; in female subjects, it is responsible for a 50.5% increase in lung cancer deaths, and a 54.8% increase in breast cancer by 2000, a change that exceeds the contribution due to population growth and ageing (Table 3).

The numbers of deaths for all cancers combined were calculated by summing the predicted numbers of deaths for the individual cancers plus ‘all other sites’. The ASRs in men are almost double those in women. Compared with 1991, the ASRs have declined a little in males (−0.8%, from 162.4 per 10^5^ to 161.1 per 10^5^), but increased slightly in female subjects (+2.5%, from 86.6 per 10^5^ to 88.8 per 10^5^) in 2005 (Table 2). The absolute numbers of deaths from all cancers are predicted to increase from 1.2 million in 1991 (752 000 in male, 422 000 in female subjects) to 1.5 million in 2000 (993 000 in male, 550 000 in female subjects), and 1.8 million in 2005 (1.1 million in male and 649 000 in female subjects) (Table 3).

Figure 2

**Figure 2:**
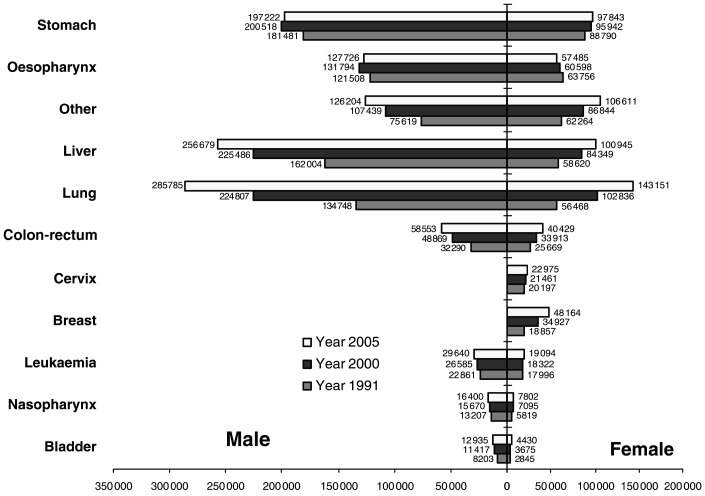
Total number of deaths for certain cancers in the years 1990, 2000 and 2005 in China, by site and sex.

summarises the predicted numbers of deaths in 2000 and 2005, relative to 1991, by site and sex. Between 1991 and 2005, the largest increase is for female breast cancer (from 19 000 deaths in 1991 to 48 000 in 2005, an increase of +155.4%), followed by lung cancer (+112.1% in men, +153.5% in women) and liver cancer (+58.4% in men, +72.2% in women).

The results of the sensitivity analysis, comparing the above results, with the three other prediction models are shown in Table 4

**Table 4:** Observed and estimated number of deaths, age-standard mortality rates with 95% estimated intervals produced by different models for four cancers in CHIS data for the year 1999

		**Number of deaths**				**Age-standard mortality (per 100 000)**			
**Cancer**	**Model**	**Observed**	**Estimated**	**95% confidence interval**		**Observed**	**Estimated**	**95% confidence interval**	
Lung	(1)	3460	3218.8	3067.7	3369.8	12.6	11.7	11.2	12.3
	(2)	3460	3217.6	3066.7	3368.5	12.6	11.8	11.2	12.3
	(3)	3460	3161.7	2906.1	3417.4	12.6	11.5	10.6	12.5
	(4)	3460	2605.3	2473.4	2777.2	12.6	9.6	9.0	10.1
									
Breast	(1)	1268	1127.6	1033.6	1221.6	4.8	4.3	3.9	4.7
	(2)	1268	1121.9	1013.5	1230.4	4.8	4.2	3.8	4.7
	(3)	1268	1104.6	957.4	1251.8	4.8	4.2	3.7	4.8
	(4)	1268	946.3	867.1	1025.4	4.8	3.6	3.3	3.9
									
Oesophagus	(1)	1690	1442.1	1328.1	1556.1	4.1	3.7	3.4	4.0
	(2)	1690	1434.9	1313.7	1556.0	4.1	3.7	3.4	4.0
	(3)	1690	1420.2	1051.6	1788.9	4.1	3.6	2.7	4.6
	(4)	1690	1413.6	1276.5	1550.8	4.1	3.6	3.3	4.0
									
Cervix	(1)	703	657.0	595.2	718.8	1.9	1.8	1.6	1.9
	(2)	703	642.4	550.5	734.3	1.9	1.7	1.4	1.9
	(3)	703	614.9	510.6	719.2	1.9	1.7	1.4	1.9
	(4)	703	619.9	514.0	725.7	1.9	1.6	1.3	1.9

, as the observed and estimated numbers of deaths (with 95% confidence intervals), and observed and estimated mortality rates (with 95% confidence intervals) in 1999. The mortality rates estimated by the selected model (1) are much closer to the observed values, with the narrowest confidence intervals, both for numbers of deaths and for the ASRs.

## DISCUSSION

Comparison of the two national mortality surveys (in 1973–1975 and 1990–1992) suggests that the second, although based on only a 10% sample of the population, was highly representative of the country as a whole (Li *et al*, 1996; Li *et al*, 1997; Yang *et al*, 2003a). It thus provides a valid baseline (for 1991) from which to project the future cancer mortality. The time trends used to project forward these rates to 2000 and 2005 derive from the CHIS routine sample survey of mortality system. In using these trends, the assumption is that they are representative of the national trends in urban and rural areas. During the study period, there were few notable changes in the population and sampling sites covered by the CHIS system. However, the geographic areas covered by the CHIS system are mainly on the eastern seaboard, the most densely populated areas of China (NOCPC, 2001), being urbanised than the national population (Rao *et al*, 1989; NCHIS, 1999). By combining estimates and predictions of age-specific and ASRs by rural and urban area, we obtained the estimates of national mortality in 2000 and 2005. The nonrepresentative nature of the CHIS sample is the probable explanation for the differences between its 1991 rates and those in the second national survey, the former being lower for certain cancers such as oesophagus and stomach and higher, in urban areas, for female breast, colon-rectum and lung. Nevertheless, it is not the values of the mortality rates in CHIS that have been used to make a national prediction, but the trends within strata of age, sex and urban–rural residence. These trends have been examined in detail elsewhere (Yang *et al*, 2003b), where it was noted that although there had been no changes in the areas covered by the sample during 1991–1999, there had been important social and demographic shifts within them, with rapid economic development and marked urban–rural migration. These will have influenced the trends observed within the urban–rural samples in addition to changes in exposures to risk factors, early detection and treatment.

Predictions of future cancer patterns use a variety of modelling strategies (Teppo *et al*, 1974; Osmond, 1985; Clayton and Schifflers, 1987; Hakulinen and Dyba, 1994; Hakulinen, 1996; Dyba and Hakulinen, 2000), their reliability depending directly on the choice of model and the explanatory variables fitted. In the absence of information on the trends in exposure to relevant risk factors, the most practical and plausible method is to use simple time-linear models on the arithmetic or logarithmic scale (Dyba and Hakulinen, 2000). Since the actual projection period in our study is short (6 years, 1999–2005), we used only the log-linear model (1), rather than models more appropriate for increasing rates that specify an identity link function (Hakulinen and Dyba, 1994; Dyba and Hakulinen, 2000). Since the Poisson assumption for the number of cases has been shown to be superior to methods that assume a normal distribution for the rates (Dyba and Hakulinen, 2000), our model specified Poisson errors for the age- and period-specific numbers of deaths, under the assumption that the observed rates of change within age groups would continue into the near future in a log-linear manner. This method closely replicated the changes observed in the CHIS mortality data set during 1991–1999 and, in the population used for the national survey in 1990–1992. However, future projections using this method may give rise to implausible distortions in the pattern of age-specific mortality for specific cancers, if the rate of change differs markedly by age group; continuation of exponential increases, in particular, can lead to ‘explosive’ forecasts if the projection period is long (Teppo *et al*, 1974). In fact, with this short projection period, the final age-specific patterns of mortality mainly appear reasonable (Table 2). Even so, some strange patterns emerge. In breast cancer, for example, age-specific mortality increases little between ages 45–54 and 75+ years, while for cervix cancer, the mortality in 2005 is highest in the age group 45–54 years (11.3 per 10^5^), decreases at ages 55–64 years (to 7.5 per 10^5^) and then rises again (to 10.9 per 10^5^ at ages 75+). These are the consequences of large rates of change in the CHIS data between 1991 and 1999 that differ by age; for breast cancer in urban female subjects, and for cervix cancer, the large increases in younger women presumably represent birth-cohort-specific changes in risk (Yang *et al*, 2003b).

Projections of cancer incidence or mortality rates based on past trends will not necessarily be accurate since they can be modified by factors that affect the risk of cancer, or its outcome; rather, projections illustrate what would have happened, if past experience continued. This in itself can be useful, as a way of establishing a baseline, or target, against which the success or failure of cancer control interventions can be evaluated (Hakulinen, 1996). Extrapolation of past trends beyond a decade or two is likely to be meaningless, and for China, in particular, the huge changes in socioeconomic situation, diet, lifestyle and environmental factors mean that all but the most short-term predictions are likely to be unrealistic. Even with our short projected period, we observe some changes that are unexpected and difficult to explain, for example, the implausible declining trend of colon-rectum cancer in rural female subjects, and the much more rapidly increasing trend of breast cancer in rural than in urban female subjects.

Declining ASRs are seen for oesophagus, stomach, cervix and nasopharynx cancers (Table 2 and Appendix A). However, demographic changes mean that the actual numbers of deaths from these cancers will increase (the population component of change exceeds the decline due to risk), except for oesophagus cancer in female subjects (Table 3). The declining ASRs are, however, largely due to declining mortality rates at age 55 years and above. At younger ages, the trend is positive for certain sites (Table 1): notably cervix cancer, especially in urban populations though decreasing in older women. Compared with the 1991 rates, those for women under 55 years were 68.5% higher in 2000, 105.0% higher in 2005, while for women over 55 years, 40.2% lower in 2000 and 56.0% lower in 2005. These age-specific trends mean that simple projection of mortality rates within age groups is likely to be misleading, and the rate of decline will certainly slow, or even reverse. The effect on the very short-term projection (6 years) in this paper is, however, likely to be small.

The remarkable changes in mortality for most major cancers, and their possible explanations have been the subject of an earlier communication (Yang *et al*, 2003b). The very large improvements in socioeconomic and sanitary conditions may be responsible for declines in cancers associated with infection and/or nutritional deficiency, such as oesophagus, stomach, cervix uteri and nasopharynx, while changes in diet and a more sedentary lifestyle, associated with an increasing prevalence of obesity, may explain some of the changes in risk for cancers of the breast and large bowel. Cigarette smoking is a risk factor for several cancers, including the lung, bladder, oesophagus, stomach, liver and cervix (IARC, 2002), and the epidemic of cigarette smoking in China is a major determinant of past and future cancer trends (Gao *et al*, 1994; Ji *et al*, 1997; CTSU, 1998; Liu *et al*, 1998).

Mortality patterns are also influenced by changes in cancer survival, which may be a consequence of earlier diagnosis, therapy or both. Some of the declines in cervix cancer mortality, most marked in urban areas, may be attributed to screening programmes. Survival from breast cancer in Tianjin increased between 1981 and 1987, and mortality rates declined, changes that were attributed to early detection programmes (Hao *et al*, 2002).

Despite the dramatic trends in mortality rates observed in the last decade, population growth and ageing are responsible for the largest component of change in the number of deaths for most cancers in 1991–2005. For most cancer sites (and for all cancers combined), the increase in the number of deaths from this component was around 50%. In the last 50 years, the Chinese population has increased dramatically, from 550 million in 1950 to 1.24 billion in 2000, and is projected to be 1.43 billion in 2020 (UN Population Division, 2003). With a declining birth rate (total fertility rate had fallen to just 1.8 children per women by 2000) and increasing expectation of life at birth, ageing will be an important characteristic of the Chinese population in the 21st century. Future population projections are based on the assumptions concerning the continuation or maintenance of such trends. Differences in the fertility or mortality assumptions could cause discrepancies in projected total population of up to 200 million or more within a 25-year projection period (UN Population Division, 2002b), although the numbers of persons in the age groups at significant risk of cancer (say 35 and above) can be fairly confidently predicted for the next 25 years. The population for 2005 used in this study was an estimate of the UN Population division (2003). Comparing this with the 2000 census shows some large discrepancies among children, e.g. 27 million more children aged 5–9 years in 2005 than children aged 0–4 years in 2000 (Figure 1), presumably due to erroneous projections of fertility. However, since most cancers occur at older ages, these errors will have very little effect on our results.

It is clear from Figure 2 that cancer will make an increasing contribution to the burden of disease in China in the 21st century. The marked increases in lung, female breast and colon-rectum cancers and the possibly underestimated increases in cervix cancer in younger women, along with the huge burden from common cancers such as oesophagus, stomach and liver, indicate the priorities for future cancer prevention and control programmes. Our projections should provide a useful benchmark against which to judge the success of cancer control work in China in the near future.

## Figures and Tables

**Figure 3:**
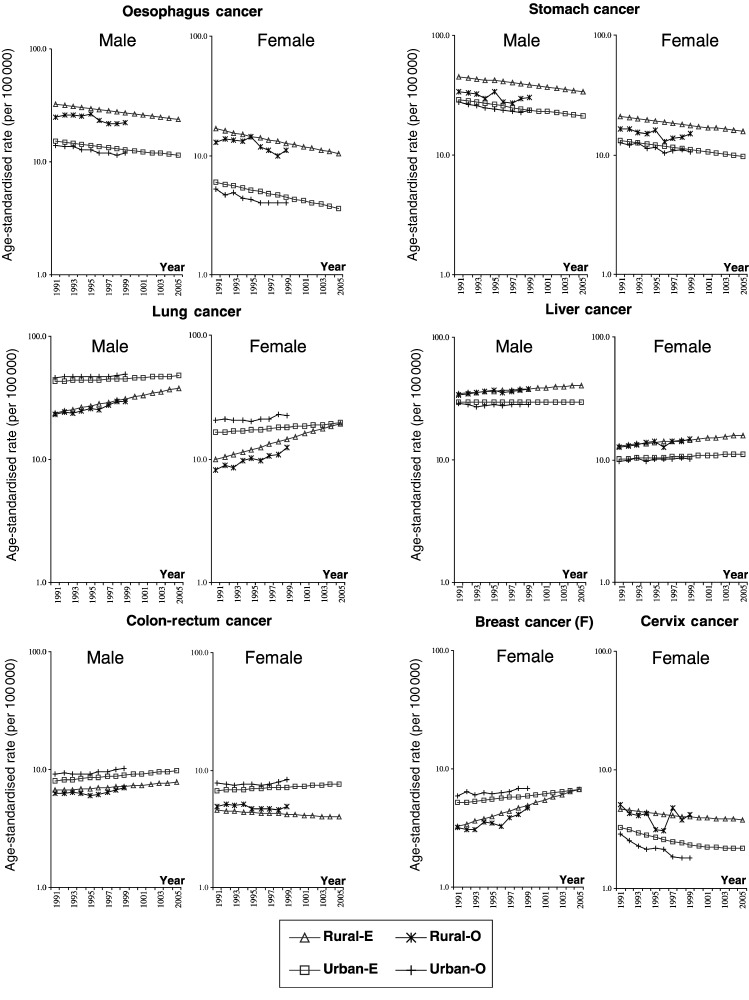
(continued)

## Appendix A

The observed, estimated and predicted age-standardised mortality rates for certain cancers in China, from 1991–2005, by site, sex and area (Figure 3
Figure 3 continued).
